# The Interplay of Cohesin and RNA Processing Factors: The Impact of Their Alterations on Genome Stability

**DOI:** 10.3390/ijms23073939

**Published:** 2022-04-01

**Authors:** Michaela Osadska, Tomas Selicky, Miroslava Kretova, Jan Jurcik, Barbara Sivakova, Ingrid Cipakova, Lubos Cipak

**Affiliations:** 1 Cancer Research Institute, Biomedical Research Center, Slovak Academy of Sciences, Dubravska cesta 9, 845 05 Bratislava, Slovakia; michaela.osadska@savba.sk (M.O.); tomas.selicky@savba.sk (T.S.); miroslava.kretova@savba.sk (M.K.); jan.jurcik@savba.sk (J.J.); 2 Institute of Chemistry, Slovak Academy of Sciences, Dubravska Cesta 9, 845 38 Bratislava, Slovakia; chembsiv@savba.sk

**Keywords:** cohesin, RNA processing factors, sororin, chromosome segregation, genome stability

## Abstract

Cohesin, a multi-subunit protein complex, plays important roles in sister chromatid cohesion, DNA replication, chromatin organization, gene expression, transcription regulation, and the recombination or repair of DNA damage. Recently, several studies suggested that the functions of cohesin rely not only on cohesin-related protein–protein interactions, their post-translational modifications or specific DNA modifications, but that some RNA processing factors also play an important role in the regulation of cohesin functions. Therefore, the mutations and changes in the expression of cohesin subunits or alterations in the interactions between cohesin and RNA processing factors have been shown to have an impact on cohesion, the fidelity of chromosome segregation and, ultimately, on genome stability. In this review, we provide an overview of the cohesin complex and its role in chromosome segregation, highlight the causes and consequences of mutations and changes in the expression of cohesin subunits, and discuss the RNA processing factors that participate in the regulation of the processes involved in chromosome segregation. Overall, an understanding of the molecular determinants of the interplay between cohesin and RNA processing factors might help us to better understand the molecular mechanisms ensuring the integrity of the genome.

## 1. Introduction

Chromosome segregation is a tightly regulated process that mediates the equal distribution of genetic material into daughter or germ cells. Defects in this process often lead to an anomalous state known as aneuploidy. Although aneuploidy is closely linked to a number of human diseases, such as infertility, miscarriages, birth defects, and cancer [1,2,3], our understanding of the molecular mechanisms leading to aneuploidy is still limited.

Cohesin plays a crucial role not only in sister chromatid cohesion but also in many other cellular processes, including DNA replication, centrosome duplication, chromatin organization, recombination, transcription regulation, and the repair of DNA damage [4,5,6,7,8,9]. There is a number of reports arguing that specific mutations in cohesin complex subunits or changes in their expression might affect the functionality of cohesin and lead to incorrect chromosome segregation and aneuploidy [10,11,12,13,14,15].

However, mutations or changes in the expression of cohesin complex subunits are not the only cause of aneuploidy. Many studies have reported that mutations in or the altered expression of spindle assembly checkpoint components also contribute to chromosome missegregation and aneuploidy [16,17,18,19,20]. Furthermore, it has been proposed that alterations in RNA processing factors or imbalances in their interactions with cohesin complex subunits might affect the fidelity of chromosome segregation and result in the genome instability phenotype [21,22].

In this review, we provide a brief overview of the cohesin complex and its role in the processes of chromosome segregation, with a special focus on the importance of RNA processing factors for proper chromosome segregation and the maintenance of genome integrity.

## 2. Mitosis and Its Pitfalls

In order to preserve the correct number of chromosomes in each cell, duplicated chromosomes have to be equally distributed into daughter cells during each cell division. Eukaryotic cells possess complex surveillance mechanisms, which promote and coordinate the processes of chromosome segregation to maintain the integrity of the genome. These include mitotic spindle checkpoints [23], the regulation of the centrosome number [24], the correction of the attachments between spindle microtubules and kinetochores [25], the regulation of sister chromatid cohesion [26], and cell cycle regulation [27].

Generally, mitosis comprises several consecutive phases. Following the replication of DNA, the kinases, such as cyclin-dependent kinase 1 (CDK1), polo-like kinase 1 (PLK1), and kinases belonging to the Aurora family (Aurora-A and -B), are activated. Their activation triggers the breakdown of the nuclear membrane and allows the condensation of chromosomes and the separation of centrosomes into opposite cell poles [28]. If mitosis is managed in a proper manner, cells enter mitosis with two centrosomes [29]. It should be noted that the deregulation of centrosome duplication during the S phase and defects in the timing of centrosome separation frequently increase the rate of incorrect kinetochore attachments, which, in turn, results in the uneven segregation of chromosomes or in the segregation of chromosomes on multipolar spindles [30]. In mammalian PtK1 cells, in which the separation of centrosomes was not completed before nuclear envelope breakdown, a higher rate of kinetochore failed attachments and chromosome missegregation was observed [24]. Therefore, the improper timing of centrosome duplication and separation associated with incomplete spindle pole separation represents a significant source of genomic instability.

Under normal conditions, the proper attachment of kinetochores to microtubules emits a signal to turn off the spindle assembly checkpoint (SAC). The SAC is a conserved mitotic regulator that is important for the metaphase-to-anaphase transition. It ensures that chromosome segregation continues only if all the kinetochores are connected to the microtubules oriented towards the opposite spindle poles [31]. Furthermore, at the onset of anaphase, it is necessary to break the cohesin rings holding together the sister chromatids. This is achieved by the activation of a multi-subunit ubiquitin ligase, the anaphase-promoting complex (APC/C). The APC/C degrades a specific cysteine protease separase inhibitor securin. At the same time, the APC/C contributes to the degradation of cyclin B, which inactivates CDK1 and allows mitotic exit and the completion of cell division and cytokinesis [32,33]. The activated separase then cleaves the cohesin subunit SCC1 (also known as RAD21) at the centromeres to open the cohesin rings, allowing the separation of the sister chromatids [34,35].

**Figure 1:**
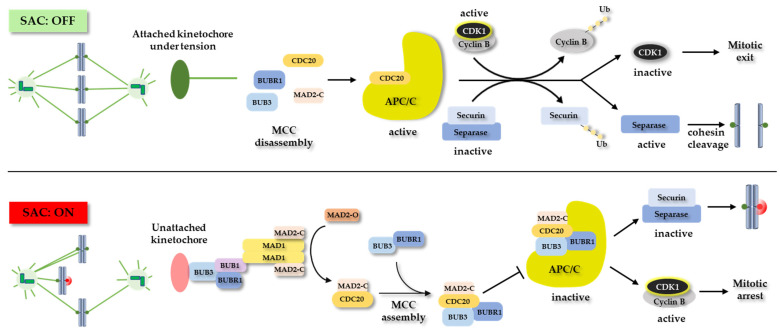
A molecular view of SAC signaling. Correct attachment of kinetochores to microtubules emanating from opposite spindle poles does not turn on the SAC (SAC: OFF). This results in MCC disassembly and activation of APC/C. Active APC/C contributes to degradation of cyclin B, which activates CDK1 and allows mitotic exit. At the same time, APC/C degrades securin, which inhibits separase. Activated separase cleaves the cohesin rings, allowing the separation of the sister chromatids. By contrast, unattached or incorrectly attached kinetochores activate the SAC (SAC: ON). This leads to formation of MCC, which binds to and inactivates APC/C. This prevents degradation of securin and cyclin B, leading to mitotic arrest.

However, improperly bound kinetochores are recognized by SAC components, which activate the SAC and catalyze the formation of the mitotic checkpoint complex (MCC). The MCC represents a complex signaling network assembly of the proteins MAD2, CDC20, BUBR1, and BUB3, with the ability to inhibit the activity of the APC/C. Activated MCC causes a delay in mitotic progression until the accurate bi-orientation of sister kinetochores is achieved, thereby preventing chromosome missegregation [36]. Interestingly, a signal from a single incorrectly attached kinetochore is sufficient to inhibit the entire sister chromatid separation [37] (Figure 1).

While the global loss of SAC function is lethal for most cells, weakened SAC activity, which could be a consequence of mutations in the proteins involved in SAC functions, leads to a precocious anaphase onset and an increased probability of incorrect chromosome segregation. The best evidence for this is a rare disease called mosaic-variety aneuploidy, in which BUB1 is mutated [38]. However, SAC mutations are very rarely found in cancer cells, and are almost invariably associated with aneuploidy. For instance, a reduced level of MAD2 was detected in breast cancer [39], and BUB1 mutations were found in prostate and in liver cancers [40,41].

Furthermore, another risk associated with the failure of SAC is mitotic error, known as merotelic attachment, in which one kinetochore of a sister chromatid pair is connected to microtubules emanating from both spindle poles [42]. Merotelic attachment occurs naturally in the early phase of mitosis and is usually corrected prior to the onset of anaphase. Since merotelic kinetochores achieve the correct number of microtubule connections, SAC does not recognize the defective kinetochore-microtubule attachment orientation. Consequently, if merotelic attachment is not corrected, lagging chromosomes occur, and equal chromosome segregation most likely fails [43]. Thus, lagging chromosomes pose a threat associated with the possibility of their entrapment and damage during cytokinesis and with the increased risk of whole-chromosome aneuploidy, as well as structural aneuploidy (structural alterations in chromosomes that include deletions, amplifications, and translocations). Therefore, merotely is considered to be a significant source of chromosome imbalances [14,44].

## 3. The Cohesin Complex and Its Role in Chromosome Segregation

The successful distribution of genetic material into daughter cells depends on cohesion between newly synthesized sister chromatid pairs. This physical linkage is known to be essential for the prevention of the premature segregation of sister chromatids and relies on an evolutionary and functionally conserved multi-subunit protein complex, known as cohesin. In addition to the function of cohesin in the regulation of sister chromatid separation [45,46], it also plays important roles in other cellular processes, such as DNA replication, DNA damage repair, the regulation of gene expression, and spatial chromosome organization [47,48,49,50,51,52,53]. Importantly, recent studies have suggested that cohesin also functions as a molecular motor, catalyzing the extrusion of DNA into the loops, and thus directly regulates genome organization [54,55,56,57,58]. Furthermore, a recent analysis of the genetic interactions between cohesin-complex-related genes and >1400 genes in the *S. cerevisiae* revealed 373 novel genetic interactions, suggesting the involvement of cohesin-related proteins in such biological processes as post-replication DNA repair, microtubule organization, and protein folding [59].

Cohesin belongs to a conserved family of the structural maintenance of chromosomes (SMC) complexes with the ability to encircle chromatin [7,60,61,62,63,64,65]. It forms a ring-shaped complex composed of four core structural subunits, SMC1, SMC3, RAD21, and stromalin antigen (SA) protein (Table 1). SMC1 and SMC3 belong to the SMC protein family, and RAD21 protein belongs to the kleisin alpha family. SMC1 and SMC3 are long, flexible coiled-coil proteins forming a V-shaped heterodimer that is tightly linked via the SMC hinge domains on the one side and globular ATP-head domains on the other side. The cohesin subunit RAD21 binds to the ATPase head, and stabilizes the interaction between the SMC1 and SMC3. SMC1, SMC3, and RAD21 thus create the tripartite ring required for the topological entrapping of DNA. Additionally, RAD21 interacts via its middle region with SA proteins (there are two paralogs, SA1 and SA2, in humans) [66]. Interestingly, both SA1 and SA2 subunits contribute to the three-dimensional genome structures besides their essential role in the maintenance of a stable association between chromatin and cohesin [67,68,69,70].

Apart from its four core structural subunits, the cohesin complex also contains several accessory and regulatory subunits, including PDS5, WAPL, sororin, and the loading-complex-forming proteins, NIPBL and MAU2. In vertebrates, PDS5 (there are two isoforms: PDS5A and PDS5B) is essential for the dissociation of cohesin complexes from the chromosome arms in early mitosis [71,72]. Another cohesin cofactor subunit, called WAPL, associates with PDS5 and, similarly to PDS5, acts as a crucial protagonist in the removal of cohesin complexes during the prophase [73,74]. Recently, it has been shown that WAPL and PDS5 also play roles in DNA replication, chromosome organization, gene expression, and DNA damage repair [75,76].

On the other hand, the loader complex subunits, which include NIPBL and MAU2, associate and create a heterodimer, which facilitates cohesin loading on chromatin [77,78,79]. The NIPBL is also supposed to be essential in cohesin translocation along chromatin [80,81,82], and was proposed to be involved in the regulation of cohesin functions [83]. Finally, sororin (cell division cycle associated 5 protein, CDCA5) is known to associate with cohesin-complex-related proteins and to contribute to sister chromatid cohesion. Sororin appears to be a regulator of the interface between cohesin and chromatin [84,85]. Importantly, a recent study of *S. cerevisiae* identified several novel proteins that also contribute to sister chromatid cohesion. These include the prefoldin complex components Gim3, Gim4, and Yke, and a microtubule-associated protein, Irc15, which is involved in the dynamics of kinetochore regulation and has an important role as a cohesin loader in centromeric cohesion establishment [59].

The loading of cohesin onto chromosomes occurs from telophase to early G1 phase [66]. The establishment of cohesin on chromatin depends on the complex of two adherin proteins, NIPBL and MAU2 [77], and also on the activity of ESCO1 and ESCO2 acetyltransferases. These acetyltransferases acetylate two lysine residues, K105 and K106, located in the SMC3 head domain, stabilizing the sister chromatid cohesion during the replication of DNA [86]. Furthermore, the acetylation of SMC3 promotes sororin binding to cohesin to mediate proper cohesion [87]. Sororin also binds to the PDS5 to compete with the cohesion-destabilizing protein WAPL [88]. The tethering of sister chromatids is maintained during the G2 phase until the metaphase-to-anaphase transition, when cohesion has to be dissolved. The removal of cohesin from chromosomes occurs in a two-step manner. Most cohesin complexes dissociate from the chromosome arms by a separase-independent pathway during prophase [89] which requires the activity of the kinases Aurora B and PLK1, as well as the cohesin subunit proteins WAPL and PDS5 [72]. Furthermore, the phosphorylation of sororin during mitosis causes its inability to antagonize WAPL binding to PDS5, which results in the association of WAPL with PDS5 and the subsequent removal of cohesin complexes from the arms of chromosomes [88].

Since most cohesin complexes dissociate from the chromosome arms, the fidelity of chromosome segregation relies on the persistence of cohesion in the centromere regions until the chromosomes are properly bi-oriented, and the SAC is satisfied. The protection of cohesion in centromeres also relies on various proteins, including shugoshin (SGO1), which is recruited to the inner centromeres with antagonizing functions against both Aurora B and CDK [90,91,92], protein kinase BUB1, and heterochromatin protein HP1α [93]. Recently, it was revealed that a mitotic histone kinase, Haspin, is essential for centromeric cohesion [94]. Haspin was shown to antagonize WAPL through binding to the YSR motif of non-catalytic N-terminus of WAPL, thereby ensuring accurate centromeric cohesion [95]. Finally, at the onset of anaphase, the remaining cohesin complexes are released from the centromeres by the cleavage of RAD21 by separase, and replicated chromosomes are pulled to the opposite spindle poles [32].

In addition to the canonical function of cohesin, which is to hold sister chromatids together from S phase until the onset of anaphase, this multiprotein complex also plays a crucial role in the DNA damage response. It was shown previously that the activation of the DNA damage checkpoint elicits cellular arrest in G2/M [96]. This is accompanied by the loading and accumulation of the cohesin complex around the sites of double-strand breaks and along chromosomes, facilitating DNA repair through favoring the use of the sister chromatid during recombination DNA repair [49,97,98,99,100]. Recently, it has been shown that there are multiple direct and indirect ways in which cohesin regulates the homology search during recombinational DNA repair and, thus, contributes to the maintenance of the structural integrity of chromosomes [101]. Additionally, recent studies have also pointed at the role of cohesin in homologous telomeric recombination, the process involved in the repair of telomeric double-strand breaks [102,103,104,105].

## 4. Cohesin Complex Defects and Diseases

The altered expression of or mutations in cohesin-related genes are often associated with chromosome segregation errors, chromosomal instability, DNA damage repair errors, or aneuploidy, which are common hallmarks of cancer and other genetic diseases [106,107,108,109].

Recently, it has been shown that the SMC3 subunit is often overexpressed in colorectal carcinoma [110], and that both RAD21 and SMC3 subunits are overexpressed in breast and prostate cancers [111,112]. Similarly, PDS5 and RAD21 were found to be significantly overexpressed in glioblastoma and gastric tumors [113,114]. The overexpression of SMC1A was also detected in hepatocellular and colorectal carcinomas [115,116].

On the other hand, the decreased expression of cohesion-related genes was linked to the development of cancer due to the effect of reduced levels of cohesin proteins on sister chromatid cohesion. A systematic analysis of 102 human homologs of 96 yeast chromosome instability genes resulted in the identification of somatic mutations in four sister-chromatid-cohesion-related genes: SMC1A, SMC3, NIPBL and STAG3 [117]. Considering this finding, the decreased expression of the cohesin subunits SMC1A, STAG2, SMC3, RAD21, cohesin loader NIPBL, and cohesin acetyltransferase ESCO1 was observed, and weakened sister chromatid cohesion, responsible for the chromosome instability phenotype, was detected in colorectal cancer [117,118].

Similarly, many other cohesin-related gene mutations were detected in various tumors, such as mutations in SA2 and STAG2, which were found in bladder carcinoma [119], Ewing sarcoma [120], and melanoma [10]. Mutations present in STAG2, SMC3, RAD21 and SMC1A were also described in acute myeloid leukemia [121,122,123,124,125]. Moreover, mutations in the SMC1A have been confirmed in early colorectal adenomas, which are thought of as a precancerous step in colon cancer development [126].

Interestingly, the recent evidence suggests that not all mutations in cohesin-related genes cause defects in sister chromatid cohesion, or lead to chromosome missegregation and aneuploidy. For example, the depletion of STAG2 in urothelial bladder cancer was not associated with an increase in aneuploidy; instead, the cells became euploid. A chromosomal copy number analysis showed that in STAG2-deficient bladder tumors, only 2 of 11 tumors displayed the loss of one copy of chromosome [21]. Moreover, a euploid karyotype common in acute myeloid leukemia was also associated with loss-of-function mutations in STAG2 [122]. Consistent with this finding, studies of naturally occurring STAG2-deficient tumors showed only a slight correlation between mutations in STAG2 and aneuploidy. It was also found that while all the tested nonsense mutations in STAG2 resulted in some reduction in sister chromatid cohesion, only one led to altered chromosome number and aneuploidy. In addition, the missense mutations in STAG2 showed unaltered cohesion. Since STAG2 is encoded on the X chromosome, its mutations should lead to the complete loss of its function [13]. However, it has been found that the entire loss of STAG2 may be partially compensated for by STAG1. Nevertheless, the loss of both STAG2 and STAG1 leads to cell death [127,128,129]. Similarly, a declining correlation between aneuploidy and direct mutations in STAG2, RAD21, SMC1A, and SMC3 was observed [120,123].

Although an increasing number of studies has correlated the defects in the cohesin complex with the formation of a wide range of cancer types, the exact genetic cause of aneuploidy in cancer cells is currently not known. Recent findings indicate that the development of aneuploidy depends on several circumstances, and is not exclusively linked to direct mutations in the cohesin subunits. Instead, it is related to an alteration in the interactions of the cohesion complex with chromatin or with gene expression regulation via DNA loop extrusion [67,130,131,132].

In addition to the above-mentioned mutations in or alterations in the expression of cohesin-related genes in cancer cells, a number of other syndromes and pathologies arise from mutations or alterations in the cohesin complex or its regulators. These syndromes and pathologies are collectively called cohesinopathies [108,133,134]. Among them, the best-defined is Cornelia de Lange syndrome (CdLS). Patients with CdLS have mutations in the gene encoding SCC2/NIPBL, a protein in the adherin complex that is required for the loading of cohesin to chromatin [135,136]. Interestingly, the NIPBL was also shown to mediate local chromatin modifications through the recruitment of histone deacetylases, but mutations in NIPBL dramatically reduced this recruitment activity [137]. Additionally, mutations in the cohesin subunits SMC1α, SMC3, and RAD21, as well as in HDAC8, have been detected in patients with CdLS [138,139,140,141]. More recently, Bromodomain-Containing 4 (BRD4), a NIPBL interactor, and Ankyrin Repeat Domain 11 (ANKRD11), an inhibitor of the ligand-dependent activation of transcription, were found to be mutated in CdLS [142]. Other cohesinopathies include Robert’s syndrome, which is a rare autosomal recessive disorder resulting from mutations in *ESCO2* [143,144], the Warsaw breakage syndrome, resulting from recessive mutations in the iron–sulfur DNA helicase (DDX11) essential for chromatid cohesion [145], and the X-linked α-thalassemia mental retardation syndrome, which is caused by the dominant mutations in *ATRX* encoding a chromatin remodeler that contributes to chromosome dynamics during mitosis [146,147].

The presence of various mutations, the altered expression of cohesin-related genes, and the phenotypes observed in cancer cells or in cohesinopathies suggest that our current understanding of cohesin’s functions is still incomplete. Further research on the molecular mechanisms that trigger cohesin’s functions, leading to pathological phenotypes, is needed.

## 5. Link between Alterations in RNA Processing Factors and Chromosome Segregation

Most eukaryotic genes undergo pre-mRNA splicing, the post-transcriptional multistep process carried out by a dynamic and evolutionarily conserved multimegadalton ribonucleoprotein (RNP) complex termed spliceosome [148]. Considering the complexity of pre-mRNA splicing, it has become clear that errors in any step of this process may lead to alterations in the open reading frames, disruptions of the protein coding sequences, the degradation of mRNAs, or the generation of non-functional proteins. Moreover, the global deregulation of pre-mRNA splicing may result in the accumulation of aberrant splice isoforms and lead to the development of many diseases [149,150]. For instance, mutations in the pre-mRNA splicing components of the U4/U6-U5 tri-snRNP lead to the development of a rare human disease called retinitis pigmentosa [151], or to a wide spectrum of craniofacial disorders [152].

Recently, genome-wide RNAi screens indicated the importance of several splicing factors for the regulation of the expression of proteins essential for cell division, especially for sister chromatid cohesion [153,154,155,156,157]. An earlier study reported that the knockdown of the splicing factors SNRPA1, SNRPB, SNW1, DHX8, DDX5, LSM6, and SART1 is followed by defects in mitotic spindle assembly [158] (Table 2). Furthermore, the depletion of CCD5L, a protein involved in the regulation of expression, as well as in the splicing of a subset of genes important in mitosis and DNA damage repair, induced mitotic arrest [159,160]. Intriguingly, point mutations in yeast HSH155, the homolog of human SF3B1, caused divergent functions in mitotic spindle through intron retention in a α-tubulin transcript of the TUB1 [161]. Another study revealed that the splicing factors Sf3A2 and PrP31 functioned directly in mitosis, independently of their function in the splicing of pre-mRNA. In *D. melanogaster*, these factors were associated with the spliceosomal B complex, and were required for the regulation of the interplay between kinetochores, spindle microtubules, and the Ndc80 complex. The depletion of Sf3A2 and PrP31 resulted in defective spindle assembly formation, metaphase arrest and aberrations in chromosome segregation [162]. Additionally, the depletion of PrP31 influenced the alternative splicing of the genes involved in mitosis and DNA repair during the early stages of embryogenesis [163].

Additionally, several studies pointed out that the accurate maintenance of sister chromatid cohesion and mitotic spindle assembly is particularly sensitive to alterations in the splicing machinery [22,153,164,165,166]. For example, the mutations in SF3B1, the gene encoding spliceosomal protein U2 small nuclear ribonucleoprotein, resulted in the inactivation of a specific regulatory subunit, PPP2R5A, of the PP2A phosphatase complex, which in turn stabilized MYC oncogene protein and exacerbated apoptosis [167]. The over-expressed MYC then promoted defects in mitotic spindle assembly, leading to delayed bi-polar spindle formation and the chromosomal instability phenotype [157].

Importantly, a recent systematic analysis revealed that cohesin might directly interact with several splicing factors and RNA-binding proteins [22]. It has been found that the splicing factors SF3B1, SF3B3, ADAR1, PRPF31, SNRNP200, EFTUD2, HNRNPU, RBM10, RBM15, HNRNPH, HSPA8, PDCD11, THRAP3, DDX47, PRPF6, and RNA-binding proteins, including the U4/U6-U5 tri-snRNP complex, physically interact with cohesin. The interactions of the splicing factors/RNA-binding proteins with cohesin were more efficient with SMC1A, SMC3, STAG2, WAPL, and PDS5A-containing cohesin complexes. Of these, the splicing factors and RNA-binding proteins appeared to interact more efficiently with PDS5A-containing cohesin complexes. It was also found that these interactions were RNA- and DNA-independent, occurred in chromatin, were enhanced during mitosis, and required RAD21. As the depletion of cohesin-interacting splicing factors or RNA-binding proteins resulted in aberrant mitotic progression, it is apparent that they must be essential for mitotic progression and proper chromosome segregation [22].

Interestingly, in recent years, many studies have reported that splicing factor depletion might impair sister chromatid cohesion through the loss of the function of a cohesin accessory subunit called sororin, suggesting that the loss of sororin might correspond to an indirect consequence of the defective splicing of its pre-mRNA [168,169,170,171,172,173].

Sororin is a basic protein with a predicted molecular size of 27 kDa. It is encoded by *CDCA5*. This gene was originally identified by a meta-analytical gene expression screen of the cell-cycle-associated transcripts co-expressed in a set of other gene transcripts with closely related functions in cell division, such as CDK1, cyclin B, and BUB1 [174]. Sororin was first detected in vertebrates, and its ortholog, called Dalmatian, was later also found in *D. melanogaster* [155].

Sororin is known as a key protein that stabilizes the interactions between cohesin and chromatin during S phase to G2 by antagonizing WAPL [77,85,88]. Both sororin and WAPL form a cohesin regulatory complex, in which they compete to bind the PDS5 [88]. Once sororin is associated with PSD5, it inhibits the function of WAPL, thereby protecting premature WAPL-mediated cohesin release from chromatin [84,87,88]. Hence, the loss of sororin immediately after DNA replication leads to a reduction in cohesin establishment on chromatin [85,88,175], and results in defects in chromosome segregation [84,87,88]. Under normal conditions, the release of sororin from chromatin is regulated by its phosphorylation by CDK1/cyclin B, PLK1 and Aurora B [176]. Once sororin dissociates from cohesin, WAPL in turn binds to PDS5, resulting in the removal of cohesin from the chromatid arms [177]. It has been suggested that sororin’s function relies on its current protein level within the cell, which is in turn associated with the stage of the cell cycle. It has been shown that sororin is present in high levels during the S phase to G2, and subsequently its level decreases at exit from mitosis [84]. It has been found that the binding of sororin to cohesin is potentiated by the acetylation of SMC3 by both acetyltransferases ESCO1 and ESCO2 [87]. Physical interactions between sororin and the cohesin complex are also mediated through the C-terminal sororin domain and the cohesin complex subunit SA2 [178]. The last 50 amino acid residues of the sororin C-terminal domain are highly conserved in all vertebrate orthologs, and were shown to play an important role in its interaction with SA2. Mutations in or the deletion of the terminal 12 amino acids of these residues destabilized the interaction between sororin and SA2, and resulted in a loss of sister chromatid cohesion and premature chromosome separation [179].

Importantly, a recent study suggested that various splicing factors, including MFAP1, SF3B1, NHP2L1, SART1, and CDC5L, are involved in the splicing of sororin. A detailed analysis of the splicing efficiency revealed the increased retention of introns 1 and 2 in transcripts of sororin in cells lacking MFAP1, NHP2L1, CDC5L, or SART1. These cells manifested an increased release of cohesin from chromatin immediately after DNA replication. In addition, the depletion of a spliceosome component, MFAP1, caused multiple mitotic defects, such as chromosome misalignment and mitotic arrest [169]. The phenotypes were similar to those observed upon the loss of sororin [85]. Hence, the failure of sororin pre-mRNA processing might be associated with decreased levels of sororin, which consequently lead to errors in mitosis, the premature separation of sister chromatids, and, finally, might cause the genome instability phenotype.

Similarly, the pre-mRNA processing factor 19 (Prp19) and the splicing factors SF3a120 and U2AF65 were shown to be involved in both the establishment of cohesion in S phase and in mitotic sister chromatid cohesion through their role in sororin pre-mRNA splicing. The defective splicing of sororin pre-mRNA due to Prp19, SF3a120, and U2AF65 depletion was also the consequence of the increased retention of sororin introns 1 and 2 [171]. Prp19 is known for its critical role in spliceosome activation and DNA damage response [180,181]. Moreover, the knockdown of Prp19 was linked to mitotic defects, such as aberrant mitotic spindles, cell arrest in prometaphase, and chromosome misalignment [171,182,183].

Cohesion deprivation consistent with a rapid reduction in mature sororin protein levels was also observed upon the knockdown of another spliceosome component—SNW1 [170]. SNW1 is a highly conserved part of an NTC-related sub-complex, which plays a crucial role in the removal of intron sequences before the mature mRNAs are exported from the nucleus to the cytoplasm [184]. RNA-seq analyses of SNW1-depleted cells revealed the retention of sororin intron 1 [170]. The loss of SNW1 caused errors in mitotic spindle assembly and affected cohesin establishment on chromatin [158]. Similarly, the correlation between the reduction in sororin levels and cohesion defects was observed after the depletion of the spliceosomal U5 subcomplex subunit PRPF8 [148,170]. Similarly, the depletion of an atypical ubiquitin-like protein, UBL5, which strongly cooperates with pre-mRNA splicing machinery components SART1 and EFTUD2, led to changes in sororin pre-mRNA splicing and expression. The depletion of UBL5 caused dysfunctional pre-mRNA splicing, which resulted in globally enhanced intron retention, including the sororin transcripts. This resulted in the defective alignment of chromosomes at the metaphase plate, delayed anaphase onset, and the premature collapse of sister chromatid cohesion [168]. The accumulation of unspliced sororin pre-mRNA, with increased retention of introns 1 and 2, was also found in hepatoma cell lines PLC/PRF/5 and HeLa cells depleted of splicing regulator SLU7 [172]. SLU7 is an evolutionarily conserved mRNA binding protein that is required in the second catalytic step of pre-mRNA splicing [185]. SLU7 deregulation caused defects in spindle assembly and cell cycle arrest, and led to the increased formation of genome-threatening RNA–DNA hybrid structures [172,186].

Defects in sister chromatid cohesion, as a result of the increased retention of sororin intron 1, were also observed for the depletion of splicing factors AQR, CRNKL1, MFAP1, NHP2L1, PRPF8, SF3B1, SNRPD2, SNRPD3, and SNRPF. In addition, an interplay between the splicing factors SNRPD2, SNRPD3, and NHP2L1 and mitotic protein SUN2, which has an important role in mitotic spindle assembly, was detected. Interestingly, the retention of sororin intron 1 was also observed upon SUN2 knockdown. Thus, it can be concluded that SUN2 might be another factor required for the proper splicing of sororin [173]. Incomplete sororin splicing, which led to reduced levels of sororin, was further detected after the depletion of cactin [165]. Cactin-depleted cells showed defects in sister chromatid cohesion, leading to premature chromosome separation and cell proliferation arrest. Cactin is known as a protein that strongly associates with the spliceosome in an effort to ensure the efficient splicing of a number of pre-mRNAs that are required in a broad range of cellular functions [165,187,188,189].

The above-mentioned findings clearly suggest that the disturbance of sororin pre-mRNA splicing leads to several mitotic aberrations, including a premature loss of sister chromatid cohesion, and results in defective chromosome segregation [169,170,173]. However, the question of how the depletion of splicing factors leads to reduced sororin levels requires further study. One can speculate that the defects in cohesion caused by the retention of intron 1 of sororin could be explained by the generation of a non-functional truncated sororin protein. This should lead to a translational frame shift and the generation of sororin transcripts with premature termination codons. Such transcripts are normally recognized and rapidly degraded by a surveillance mechanism called nonsense-mediated RNA decay (NMD) [190]. However, recent findings have suggested that the NMD pathway is attenuated when premature termination codons are located very far downstream of the last exon junction complex, as well as in short-half-life pre-mRNA [191,192], which is the case of sororin pre-mRNA. Additionally, sororin became degraded during each mitotic exit, and it had to be de novo synthesized during every S phase [84].

**Table 2:** Summary of RNA processing factors whose deregulation affects fidelity of chromosome segregation and genome stability.

Organisms	RNA Processing Factors	Phenotypes
*H. sapiens*	SNRPA1, SNRPB, SNW1, DHX8, DDX5, LSM6, SART1	Defects in mitotic spindle assembly [158]
*H. sapiens*	SF3B1, SF3B3, ADAR1, PRPF31, SNRNP200, EFTUD2, HNRNPU, RBM10, RBM15, HNRNPH, HSPA8, PDCD11, THRAP3, DDX47, PRPF6	Aberrant mitotic progression [22]
*H. sapiens*	MFAP1, SF3B1, NHP2L1, SART1, CDC5L	Premature cohesin release from chromatin, chromosome misalignment, mitotic arrest [169]
*H. sapiens*	Prp19, SF3a120, U2AF65	Aberrant mitotic spindles, cell cycle arrest in prometaphase, chromosome misalignment [171,182,183]
*H. sapiens*	SNW1	Errors in mitotic spindle assembly, affected cohesin establishment on chromatin [158,170]
*H. sapiens*	UBL5	Defective alignment of chromosomes at the metaphase plate, delayed anaphase onset, prematured collapse of sister chromatid cohesion [168]
*H. sapiens*	SLU7	Defects in spindle assembly, cell cycle arrest, increased formation of R-loops [172]
*H. sapiens*	AQR, CRNKL1, MFAP1, NHP2L1, PRPF8, SF3B1, SNRPD2, SNRPD3, SNRPF	Defects in sister chromatid cohesion [173]
*D. melanogaster*	Sf3A2, PrP31	Defective spindle assembly formation, metaphase arrest, aberrations in chromosome segregation [162]
*S. cerevisiae*	HSH155 (homolog of human SF3B1)	Divergent functions of mitotic spindle through intron retention in α-tubulin transcript of the TUB1 [161]

## 6. Conclusions

In summary, it can be postulated that under specific circumstances, mutations in core or accessory cohesin subunits, or their altered expression due to alterations in RNA processing factors, including changes in the interactions between cohesin and RNA processing factors, have both direct and indirect impacts on cohesin’s functions, the segregation of chromosomes, and genome stability. Interestingly, there seems to be a strong correlation between the regulation of sororin protein levels and the dysfunctionality of RNA processing factors, which strongly affect the dynamics of sister chromatid cohesion.

Although much progress has been made in understanding cohesin’s biology and RNA processing, further studies are required to advance the field. Deciphering the molecular determinants of the interplay between cohesin and RNA processing factors will provide a better understanding of the role of cohesin subunits and RNA processing factors for the processes of chromosome segregation and for the maintenance of genome integrity.

## Figures and Tables

**Table 1:** Cohesin subunits and cohesin regulators.

	*H. sapiens*	*D. melanogaster*	*S. cerevisiae*	*S. pombe*
Structural maintenance of chromosomes	SMC1α	Smc1	Smc1	Psm1
	SMC3	Cap/Smc3	Smc3	Psm3
	SMC1β *			
α-kleisin	RAD21	DRad21	Mcd1/Scc1	Rad21
	REC8 *, RAD21L *		Rec8 *	Rec8 *
Stromalin/HEAT repeat domain	STAG1, STAG2	DSA1	Scc3/IRR1	Psc3
Adherin (cohesin loading)	NIPBL/SCC2/Delangin	Nipped-B	Scc2	Mis4
			Scc4	Ssl3
Regulator of cohesin maintenance	PDS5A	Pds5	Pds5	Pds5
	PDS5B/AS3/APRIN			
Acetyltransferase (cohesin establishment)	ESCO1	Eco/Deco	Eco1/Ctf7	Eso1
	ESCO2	San		
Deacetylase	HDAC8		Hos1	

* Meiosis-specific cohesin subunits.

## Data Availability

Not applicable.
